# Testosterone regulates cell proliferation in aggressive fibromatosis (desmoid tumour)

**DOI:** 10.1038/bjc.2011.107

**Published:** 2011-04-05

**Authors:** H Hong, P Nadesan, R Poon, B A Alman

**Affiliations:** 1 Program in Developmental and Stem Cell Biology, Hospital for Sick Children, Toronto Medical Discovery Tower, 101 College Street, Toronto, ON, Canada M5G 1L7; 2 Department of Surgery, University of Toronto, 101 College Street, Toronto, ON, Canada M5G 1L7

**Keywords:** aggressive fibromatosis, testosterone, *β*-catenin, mouse model, APC

## Abstract

**Background:**

Aggressive fibromatosis (desmoid tumour) is a locally invasive tumour caused by mutations resulting in *β*-catenin protein stabilisation. *Apc1638N* mice are predisposed to developing aggressive fibromatosis tumours, and male mice develop greater numbers of tumours than female mice, suggesting a role for androgens in this tumour type.

**Methods:**

Human aggressive fibromatosis tumours were examined for the expression of the androgen receptor, and primary human tumour cell cultures were treated with testosterone. Orchidectomised *Apc1638N* mice were investigated for the development of tumours, and were treated with testosterone to study the effect of tumour formation and the level of *β*-catenin.

**Results:**

Androgen receptors are universally expressed in human aggressive fibromatosis tumours. Testosterone increased the proliferation rate and *β*-catenin protein level in a dose-dependent manner in human aggressive fibromatosis tumours. Orchiectomy reduced the number and size of tumours that formed in male *Apc1638N* mice to a similar level as observed in female mice. Testosterone treatment increased the number of tumours that formed in orchidectomised male mice, and resulted in a marked increase in *β*-catenin protein levels.

**Conclusion:**

Testosterone regulates *β*-catenin protein level and proliferation rate in this mesenchymal tumour. This work identifies the therapeutic use of testosterone blockade in aggressive fibromatosis as an area for further investigation.

Aggressive fibromatosis (also known as desmoid tumour) is a locally invasive fibroproliferative neoplasm that causes deformity, morbidity and occasional mortality due to local effects on vital structures. This tumour can occur as sporadic lesion or as a manifestation of the preneoplastic conditions familial infiltrative fibromatosis or familial adenomatous polyposis. Most cases of sporadic tumours are associated with somatic mutations in genes coding for *β*-catenin or APC, whereas familial cases are associated with germline mutations in the *APC* gene (Alman *et al*, 1997; Tejpar *et al*, 1999). Despite differences in specific mutations in individual cases, all aggressive fibromatosis tumours are characterised by *β*-catenin protein stabilisation and activation of *β*-catenin-mediated TCF-dependent transcriptional activity (Alman *et al*, 1997; Cheon *et al*, 2002).

*β*-Catenin is an important mediator in the canonical Wnt signalling pathway. Wnt ligands bind a receptor complex comprised of a member of a seven-transmembrane-domain receptor of the frizzled family and an LRP5/6 co-receptor (Bhanot *et al*, 1996; Pinson *et al*, 2000). In the absence of activating Wnt ligands, *β*-catenin becomes phosphorylated at serine and threonine sites encoded in exon 3, and the protein is targeted for ubiquitin-mediated degradation (Rubinfeld *et al*, 1996; Aberle *et al*, 1997). In the presence of an activating ligand, *β*-catenin is not phosphorylated at these sites, accumulates in the cytoplasm and translocates into the nucleus (Daniels and Weis, 2005). Together with TCF/LEF transcription factors, it regulates the expression of cell-type specific target genes (Behrens *et al*, 1996; van de Wetering *et al*, 1997).

A role for sex hormones in aggressive fibromatosis is suggested by data showing that tumours express receptors for sex steroid hormones (Ishizuka *et al*, 2006). In addition, male *Apc1638N* mice, which express a mutation in the *Apc* gene similar to that in familial infiltrating fibromatosis, develop substantially more tumours than female mice (Smits *et al*, 1998; Poon *et al*, 2001a), implicating androgens in this tumour. Androgens are known to have a role in the progression of prostate cancer (Gelmann, 2002; Verras and Sun, 2006), in which a crosstalk between the androgen receptor and canonical Wnt pathways has been identified at several levels in these signalling pathways (Truica *et al*, 2000; Feldman and Feldman, 2001; Balk, 2002; Mulholland *et al*, 2002; Chesire and Isaacs, 2003). Such a possibility could also exist in aggressive fibromatosis. Here, we examine the role of testosterone in aggressive fibromatosis, in both human tumours and in the *Apc1638N* mouse.

## Materials and methods

### Human aggressive fibromatosis tumour samples

Informed consent was obtained for the use of tumour material and patient data. Patients with musculoskeletal tumours treated at the University of Toronto affiliated hospitals were registered in a database that includes demographic information and pathology data. In many cases, cryopreserved tumour tissue is also available. For preparation of cryopreserved tissues for storage in the tumour bank, tissues are snap frozen as soon as possible following surgical excision. In six patients with aggressive fibromatosis tumours, cells were also collected from the tumours immediately after surgery to establish primary cell cultures. The cultures were initially established in DMEM supplemented with 10% fetal bovine serum and maintained at 37°C in 5% CO_2_ as previously reported (Alman *et al*, 1997; Li *et al*, 1998). Cells were divided when confluent and experiments were performed only between the first and third passages. Further experiments were performed in either 2% (low) or 5% (high) fetal bovine serum.

### Testosterone treatment of cell cultures

Before experimental studies, 2 × 10^4^ cells were seeded overnight in charcol-treated androgen-free culture media (Clonetech, Mountain View, CA, USA). Between 0 and 100 *μ*g ml^−1^ of dihydrotestosterone (DHT, Sigma, St Louis, MO, USA) were added to the media the next day, and cells studied for the effect of testosterone after 24 h. Cells were grown in either 5% or 2% fetal calf serum (high or low serum). Cell viability was measured using the Trypan Blue dye exclusion method, with the number of live and dead cell counted in three culture dishes for each patient sample. Proliferation was measured using bromodeoxyuridine (BrdU) incorporation with the BrdU added for a period of 12 h before analysis, and apoptosis measured using the TUNEL assay, as previously reported (Li *et al*, 2001). The percent BrdU incorporation and number of TUNEL stained cells were counted over 10 high-powered fields from three independent culture flasks for each patient sample.

### Orchidectomy and testosterone treatment of *Apc1638N* mice

Our local animal care committee approved all of the mouse studies. The generation and phenotype of *Apc1638N* mice was previously reported (Smits *et al*, 1998). Male *Apc1638N* mice underwent surgical orchidectomy or a sham procedure at 6 weeks of age. Mice were divided into four study groups, each containing 12 mice: sham non-castrated with no treatment; castrated with no treatment; castrated with vehicle control; and castrated treated with testosterone (Depo-testotsterone, Pharmacia, Saint-Laurent, QC, Canada) at 50 *μ*g per g of body weight. The drug, or carrier, was administered by intra-muscular injections every 14 days. A smaller cohort of six *Apc1638N* mice that underwent orchidectomy, were treated with 20 *μ*g per g of body weight of Depo-testosterone. Mice were examined until they were 5 months of age. At the time of autopsy, aggressive fibromatosis tumours and gastrointestinal polyps were scored for number and volume as previously reported by an observer blinded to the treatments (Smits *et al*, 1998; Poon *et al*, 2001b). Tumours and normal tissue were collected for protein extraction. A commercially available testosterone immunoassay (R&D Systems, Minneapolis, MN, USA) was used to measure the serum testosterone level in the treated and control mice.

### Expression analysis

RNA expression for the androgen receptor was carried out using real-time RT–PCR using previously reported primers and conditions, using the expression of *GAPDH* as a loading control (Linja *et al*, 2001) using RNA from 24 sporadic aggressive fibromatosis tumours. We also examined protein lysates from 18 of the human tumours, of which protein lysates were available for analysis. Western blotting was carried out using an androgen receptor antibody (Abcam ab45089, Cambridge, UK) using previously reported techniques (Lin *et al*, 2003). An antibody to GAPDH was used on the same membranes as a loading control (mouse monoclonal, Upstate Biotechnology, Lake Placid, NY, USA). Densitometery was performed for the bands using the AlphaEaseFC software (Alpha Innotech, San Leandro, CA, USA). The level of expression was compared between samples from male and from female patients. Western analysis for *β*-catenin was carried out using a mouse polyclonal antibody (Upstate Biotechnology), and Horseradish peroxidase-tagged secondary antibodies and Enhanced ChemiLuminescence (Amersham, Little Chalfont, UK) were used to detect hybridisation (Linja *et al*, 2001). *β*-Catenin expression at the RNA level was also analysed using real-time RT–PCR using previously reported primers and conditions (Goyal *et al*, 2008).

### Statistical analysis

The means, standard deviations and 95% confidence intervals were calculated for each data set. Data in the manuscript are reported as the mean and ±95% confidence intervals. Studies were performed in at least triplicates to ensure reproducibility, and data means compared using the two-tailed *t*-test as calculated using Microsoft Excel (Microsoft, Redmond, WA, USA).

## Results

### Androgen receptors are expressed in aggressive fibromatosis tumours from both males and females

**Figure 1:**
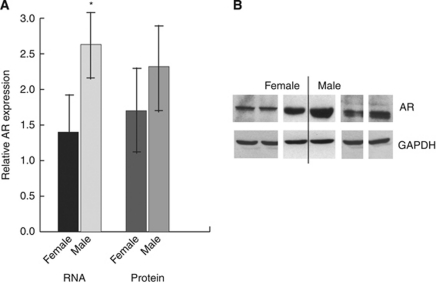
Human aggressive fibromatosis tumours express the androgen receptor. (**A**) Real-time RT–PCR (RNA) and western analysis (protein) results show that the androgen receptor is expressed in aggressive fibromatosis tumours from both men and women. At the RNA level, there is a significantly higher level of expression in tumours from males. Data are given as means and 95% confidence intervals for different concentrations of dihydrotestosterone. An asterisk above data shows a significant increased level of expression between tumours from males compared with the level of expression from tumours in females. (**B**) Representative western blots showing expression of the androgen receptor in aggressive fibromatosis tumours from both men and women, with a higher level of expression in the tumours from male patients.

We examined RNA from 24 aggressive fibromatosis tumours and protein from 18 of the tumours for expression of the androgen receptor by real-time RT–PCR or western blot analysis using previously reported techniques (Linja *et al*, 2001; Lin *et al*, 2003). All of the tumours were sporadic lesions, and half were from women. We found that all 24 cases expressed the androgen receptor. There was a significantly higher level of androgen receptor mRNA level in the tumours from males compared with tumours from females. Although there was higher mean level of the androgen receptor protein in male patients, because of the high level of variability in the protein levels, this did not reach statistical significance (Figure 1). There was not an obvious correlation between the level of expression and other clinical characteristic.

### Testosterone regulates cell proliferation aggressive fibromatosis

**Figure 2:**
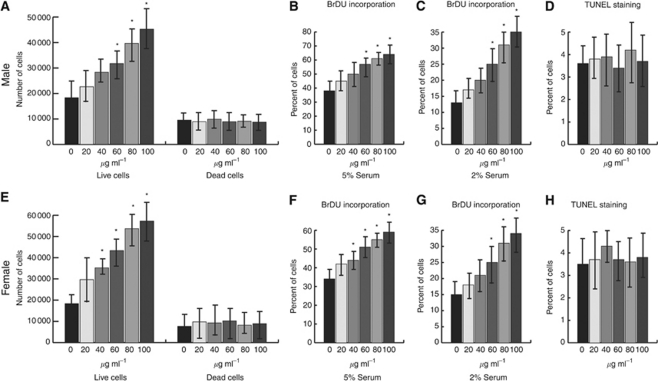
Dihydrotestosterone regulates cell proliferation in aggressive fibromatosis. Data from primary cell cultures of tumours from male patients (**A**–**D**) and female patients (**E**–**H**) show that testosterone regulates cell proliferation. (**A** and **E**) Number of dead and live cell after dihydrotestosterone (DHT) treatment. (**B**, **C**, **F** and **G**) Proliferation rate given as the proportion of cells exhibiting BrDU incorporation, (**B** and **F**) data from high serum conditions and (**C** and **G**) data from low serum conditions. (**D** and **H**) Apoptosis as detected by the proportion of cells exhibiting positive TUNEL staining. Data are given as means and 95% confidence intervals for different concentrations of dihydrotestosterone. An asterisk above data shows a statistically significant difference from control conditions. There was an increase in the number of viable cells and proliferation rate, but no change in number of dead cells or apoptosis rate with increasing doses of dihydrotestosterone. Proliferation in cell cultures from tumours from both male and female patients are responsive to dihydrotestosterone, and while there is a trend towards a greater increase in proliferation in tumours from men than from women, this did not reach statistical significance.

Despite the knowledge that androgen receptors are expressed in aggressive fibromatosis tumour cells, it is not known if androgens have a functional role in this tumour type. Thus, we examined if testosterone regulates cell behaviour in aggressive fibrmatosis, using primary cell cultures from six human tumours (three from men and three from women). Cultures were prepared as previously reported (Li *et al*, 1998), and examined in charcol-treated androgen-free culture media. Between 0 and 100 *μ*g ml^−1^ of DHT was added to the media, and cell viability, proliferation rate, apoptosis rate examined. There was a dose-dependent increase in the proliferation rate and number of viable cells with increasing DHT levels in the cell cultures but no change in number of dead cells or apoptosis rate. This finding was observed in cells grown in either high or low serum concentrations, but the relative difference was greater in cells grown in lower serum concentrations (Figure 2). There was a greater increase in cell proliferation in cell cultures maintained in low serum, and there was a greater increase in proliferation in cultures from male patients than from female patients.

### Castrated *Apc1638N* mice develop fewer and smaller aggressive fibromatosis tumours

**Figure 3:**
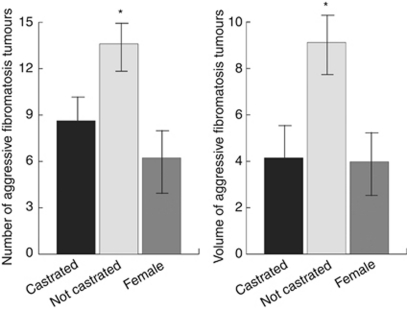
Fewer and smaller aggressive fibromatosis tumours develop in castrated *Apc1638N* mice. The number and volume of aggressive fibromatosis tumours in 5-month-old *Apc1638N* mice. Volume is given as mean mm^3^ per individual tumour. Castrated mice develop a similar number of tumours as female mice that are also of a similar volume. Data are given as means and 95% confidence intervals. An asterisk above data shows a significant difference from castrated male mice.

To examine the role of testosterone *in vivo*, we examined castrated male *Apc1638N* mice and compared the tumour phenotype to animals that had undergone a sham operation, and to female mice. The number and size of tumours that developed in 5-month-old castrated *Apc1638N* mice was significantly reduced and smaller in volume as compared with sham non-castrated mice (Figure 3). Interestingly, the number and size of tumours that developed in male castrated mice is similar to the number that developed in female mice (8.63±2.25 *vs* 6.23±2.96 average number of tumours). In contrast, there was no significant difference in the number of gastrointestinal polyps that developed in these mice between the two groups. This data suggest an *in vivo* role for androgens in aggressive fibromatosis.

### Testosterone regulates the number and size of aggressive fibromatosis tumours in castrated *Apc1638N* mice

**Figure 4:**
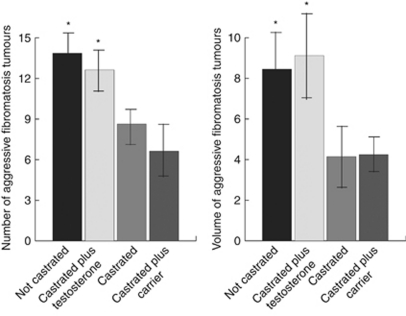
Testosterone regulates the size and number of aggressive fibromatosis tumours in castrated mice. The number and volume of aggressive fibromatosis tumours in 5-month-old *Apc1638N* mice. Volume is given as mean mm^3^ per individual tumour. Testosterone treatment brings the number and volume of tumours that develop close to that of mice that are not castrated. Data are given as means and 95% confidence intervals for different concentrations of testosterone. An asterisk above data shows a significant difference from castrated male mice.

There are a number of factors that are altered with castration, and to determine whether testosterone is a major factor contributing to the development of aggressive fibromatosis, castrated male *Apc1638N* mice were administered testosterone to restore serum levels comparable to that of control mice. We found that treatment with 50 *μ*g per g of body weight of testosterone resulted in a significant increase in the number of tumours that formed, close to that of uncastrated mice (Figure 4). An additional cohort of mice were castrated and treated with a carrier, and there was no significant difference in the number of tumours that formed compared with castrated mice alone (6.63±2.16 *vs* 8.63±2.25). The average volume per tumour in orchidectomised *Apc1638N* mice treated with testosterone was larger than those treated with carrier (Figure 4), and the average tumour volume in castrated mice was close to that in female mice (4.14±2.57 *vs* 4.15±2.08 mm^3^). Castrated mice treated with 20 *μ*g per g of body weight of testosterone developed a number and volume of tumours intermediate between the other groups. Plasma testosterone levels were 398±95 pg ml^−1^ (mean±95% confidence interval) in control mice, 6±6 pg ml^−1^ in castrated mice, 362±64 in mice treated with 50 *μ*g per g of body weight of testosterone and 178±59 pg ml^−1^ in mice treated with 20 *μ*g per g of body weight of testosterone. There was a significant difference in levels between castrated mice and mice treated with 20 *μ*g per g of body weight of testosterone compared with control mice (*P*<0.05), but there was no significant difference between mice treated with 50 *μ*g per g of body weight of testosterone and control mice. This data show that the effect of castration in the aggressive fibromatosis phenotype can be rescued by testosterone.

### Testosterone modulates *β*-catenin levels in aggressive fibromatosis tumours

**Figure 5:**
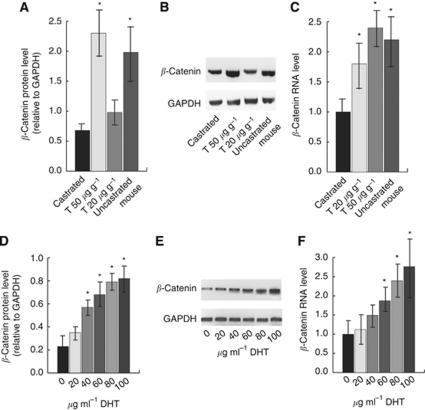
Testosterone modulates *β*-catenin levels in aggressive fibromatosis tumours. Testosterone treatment upregulates *β*-catenin protein levels in mouse and human tumours. (**A**–**C**) Data from castrated mice; (**D**–**F**) data from the human tumour cell cultures. (**A**) Relative *β*-catenin protein level (compared with GAPDH) is given for 10 mouse tumours in each group. Data are given as means and 95% confidence intervals for mice treated with low or higher dose testosterone or with carrier. (**B**) A representative western blot for four individual tumours, one from mice from each group, showing that testosterone regulates *β*-catenin protein level. (**C**) Relative *β*-catenin RNA level (compared with *Gapdh*), with castrated mice defined at ‘1’ is given for 10 mouse tumours in each group. Data are given as means and 95% confidence intervals for mice treated with low or higher dose testosterone or with carrier. (**D**) Relative *β*-catenin protein level (compared with GAPDH) from the human tumour cell cultures with various levels of testosterone treatment. (**E**) A representative western blot for cell cultures from a single tumour treated with different doses of dihydrotestosterone. (**F**) Relative *β*-catenin RNA level (compared with *GAPDH*), with control cultures defined as ‘1’ is given for the same cell cultures as in **D**. Data are given as means and 95% confidence intervals for different concentrations of dihydrotestosterone. An asterisk above data shows a significant difference from control conditions. ‘T’ indicates testosterone and ‘DHT’ indicates dihydrotestosterone.

To investigate whether *β*-catenin levels are modulated by testosterone in tumours *in vivo*, we studied tumours from *Apc1638N* mice. Tumours from castrated mice treated with carrier or with testosterone were collected for RNA and protein extraction. Western blot analysis using an antibody against total *β*-catenin demonstrated a three-fold increase in the amount of *β*-catenin in castrated mice treated with testosterone compared with those treated with carrier (Figures 5A and B). There was also a significant increase in *β*-catenin protein level in tumours from mice treated with testosterone compared with control mice (Figure 5C). We then examined the level of *β*-catenin in the six primary cell cultures from the human tumours, and found a dose-dependent increase in *β*-catenin with higher levels of DHT (Figures 5D and E).

### The frequency of sporadic aggressive fibromatosis is the same in men and women

Data on all patients treated at the University of Toronto teaching hospitals are recorded in a patient registry. We reviewed new patients who had a pathology proven diagnosis of sporadic aggressive fibromatosis that presented over the 10-year period from 1999 to 2009. There were 56 male and 48 female patients. Thus, in our population, there was not a predominance of this tumour type in either women or men at our institution. As this is the only regional referral centre for musculoskeletal tumours in central Ontario, it is unlikely that a bias due to referral patterns skewed this data.

## Discussion

Although receptors for both female and male sex hormones are present on aggressive fibromatosis tumours, most studies on the role of sex hormones in this tumour type focus on oestrogen. We found an important role for testosterone as a regulator of cell proliferation in aggressive fibromatosis, in tumours derived from both men and women. Castration of male *Apc1638N* mice substantially reduced the number and size of tumours they developed, a change that was reversed when the mice were treated with testosterone, thus showing an *in vivo* role for androgen hormones in this tumour type.

Androgens are well known to influence cell behaviour in prostate cancer, and a role for testosterone in *β*-catenin signalling in prostate cancer is suggested by data showing that androgen receptor activation potentiates Wnt signalling (Schweizer *et al*, 2008). Although little is known about the role of testosterone in mesenchymal tumours, androgens are known to affect mesenchymal cell development. Androgens promote anabolism in the musculoskeletal system, repressing adiposity. Studies in rodents show that the mechanism by which these mesenchymal cell changes occurs is mediated by cell signalling pathways that alter their differentiation potential. In mesenchymal multipotent C3H 10T1/2 cells, testosterone promotes nuclear translocation of an androgen receptor–*β*-catenin complex, thus showing that *β*-catenin activity can be regulated by testosterone in this cell type. This was also demonstrated *in vivo*, wherein testosterone level positively regulated *β*-catenin transcriptional activity in normal mesenchymal cells. Indeed, this regulation is thought to be partially responsible for the observed changes in muscle behaviour with aging, when testosterone levels decline (Singh *et al*, 2006, 2009; Gentile *et al*, 2010). *β*-Catenin also has an important role in cutaneous wound repair regulating the number of dermal fibroblasts present, and thus scar size (Cheon *et al*, 2005, 2006). The decrease in skin wound-healing rate in the elderly can be modulated by testosterone, and is characterised by smaller numbers of dermal fibroblasts in the wound. This process is also associated with a decreased level of *β*-catenin, and testosterone treatment increases the level of *β*-catenin in wound repair (Gilliver *et al*, 2009). Thus, there is evidence that testosterone regulates cell behaviour and *β*-catenin in a variety of mesenchymal cell types. Our finding that testosterone regulates *β*-catenin in this mesenchymal tumour, suggests that this is a common mechanism in a variety of normal and pathological mesenchymal cells.

As both oestrogen and testosterone have roles in modulating cell behaviour in aggressive fibromatosis, it is possible that sex steroid hormone modulation can be used to treat this tumour type. The effective use of oestrogen blockade has been reported in this tumour type, but not all tumours are responsive (Bus *et al*, 1999; Altomare *et al*, 2010). A poor outcome with oestrogen blockade in some aggressive fibromatosis tumours could be related to the role of testosterone in this tumour type. It may be that effective therapy based on sex steroid hormone modulation requires blockade of both oestrogen and testosterone. This is an area for future investigation.

The oestrogen receptors responsiveness in aggressive fibromatosis tumours has been thought to be responsible for a predominance of this tumour type in females. However, although some studies suggest a female predominance of this tumour type (Reitamo *et al*, 1986; Fallen *et al*, 2006; Dafford *et al*, 2007), others do not (Pignatti *et al*, 2000; Lefevre *et al*, 2008; Nieuwenhuis *et al*, 2008). We found a similar rate in both males and females at our centre. In addition, studies reporting on larger number of cases reporting a female predominance show only a relatively mild if any effect of gender. Thus, oestrogen responsiveness of tumour cells is unlikely to result in an overwhelming female predominance to the development of aggressive fibromatosis. This clinical information, in concert with experimental data suggest that sex steroid hormones primarily has a predominant role regulating tumour cell proliferation, rather than in the initiation of tumour development. This concept is supported by our finding that both female and male mice develop tumours. The higher level of expression of the androgen receptor in tumours from male patients and the greater responsiveness to testosterone in tumours from men suggests that the larger size of tumours in male mice is related to this responsiveness. Despite this difference, both tumours from men and women express the androgen receptor, and exhibit a proliferative rate that is responsive to testosterone. Although the mice that develop aggressive fibromatosis tumours are models for the human disease they do not necessarily recapitulate every facet of the disease. The more severe phenotype in male mice than female mice does not seem to be present in human tumours. Despite this, the *Apc1638N* mouse remains a useful animal in which to study signalling pathways involved in this tumour type *in vivo* (Poon *et al*, 2001b), but these differences highlight the importance of verifying the findings in tissue or cell cultures from patients.

*β*-Catenin is involved in a number of cell processes, including the structure of cell membrane complexes, transcriptional regulation, altering cell proliferation and regulating apoptosis (Heasman *et al*, 1994; Sanson *et al*, 1996; Gumbiner, 2000; Jamora and Fuchs, 2002; Ferenc *et al*, 2009). In aggressive fibromatosis, it has a predominant role regulating cell proliferation, an finding supported by our observation that testosterone levels correlated both with *β*-catenin levels and cell proliferation. As aggressive fibromatosis is a locally invasive tumour, slowing cell proliferation may be an effective approach to patient management, and our data raises the potential that testosterone modulation can be used to achieve this goal.

Our data extend the role of androgens to a mesenchymal tumour, aggressive fibromatosis. It explains the observation that male *Apc1638N* develop larger number of tumours, and suggests that androgen receptor-blocking agents could serve as a novel therapeutic approach to aggressive fibromatosis. Perhaps individualised therapy based in part on the relative expression of the specific sex steroid hormone receptor will prove efficacious.
